# Effects of a liquid high-fat meal on postprandial lipid metabolism in type 2 diabetic patients with abdominal obesity

**DOI:** 10.1186/s12986-017-0211-5

**Published:** 2017-08-14

**Authors:** Feng Wang, Huixia Lu, Fukang Liu, Huizhen Cai, Zhixiu Song, Fei Guo, Yulan Xie, Guofang Shu, Guiju Sun

**Affiliations:** 1 Key Laboratory of Environmental Medicine and Engineering of Ministry of Education, and Department of Nutrition and Food Hygiene, School of Public Health, Southeast University, Nanjing, China; 2 Zhongda Hospital, Southeast University, Nanjing, China; 3 School of Public Health, Ningxia Medical University, Yinchuan, China; 4 Second Clinical Medical College, Nanjing University of Traditional Chinese Medicine, Nanjing, China

**Keywords:** Type 2 diabetes, Abdominal obesity, Liquid high-fat meal, Lipid metabolism, PvuII polymorphisms

## Abstract

**Background:**

Postprandial lipemia and lipoprotein lipase (LPL) activity play crucial roles in the pathogenesis of accelerated atherosclerosis. This study aimed to evaluate the postprandial lipid metabolism after the ingestion of a liquid high-fat meal in type 2 diabetic patients with abdominal obesity, and determine if the PvuII polymorphisms of LPL influence their postprandial lipid responses.

**Methods:**

Serum glucose, insulin, triglycerides (TG), total cholesterol (TC) and high density lipoprotein cholesterol (HDL-C) were measured in fasting and postprandial state at 0.5, 1, 2, 4, 6 and 8 h after a liquid high-fat meal in 51 type 2 diabetic patients with abdominal obesity, 31 type 2 diabetic patients without abdominal obesity and 39 controls. Their PvuII polymorphisms of LPL were tested in fasting.

**Results:**

Type 2 diabetic patients with abdominal obesity had significantly higher postprandial areas under the curve (AUC) of glucose [least square mean difference (LSMD) = 30.763, 95% confidence interval (CI) = 23.071–38.455, *F* = 37.346, *P* < 0.05] and TC (LSMD = 3.995, 95% CI = 1.043–6.947, *F* = 3.681, *P* < 0.05) than controls. Postprandial AUCs for insulin, homeostasis model assessment-insulin resistance (HOMA-IR) and TG were higher (LSMD = 86.987, 95% CI = 37.421–136.553, *F* = 16.739, *P* < 0.05; LSMD = 37.456, 95% CI = 16.312–58.600, *F* = 27.012, *P* < 0.05; LSMD = 4.684, 95% CI = 2.662–6.705, *F* = 26.158, *P* < 0.05), whereas HDL-C AUC was lower (LSMD = −1.652, 95% CI = −2.685 – -0.620, *F* = 8.190, *P* < 0.05) in type 2 diabetic subjects with abdominal obesity than those without abdominal obesity. In type 2 diabetic patients with abdominal obesity, postprandial TG AUC was lower in P−/− than in P+/− (LSMD = −4.393, 95% CI = −9.278 – -0.491, *F* = 4.476, *P* < 0.05) and P+/+ (LSMD = −7.180, 95% CI = −12.319 – -2.014, *F* = 4.476, *P* < 0.05) phenotypes. Postprandial AUCs for glucose, insulin, HOMA-IR, TC and HDL-C were not different according to PvuII phenotypes.

**Conclusions:**

Abdominal obesity exacerbates the postprandial lipid responses in type 2 diabetic patients, which partly explains the excess atherogenic risk in these patients. In addition, the presence of P+ allele could contribute to a greater postprandial TG increase in type 2 diabetic patients with abdominal obesity.

**Trial registration:**

ChiCTR-IOR-16008435. Registered 8 May 2016.

## Background

It is generally known that individuals were in the state of not fasting during most of the time. Determination of blood lipids, however, is mostly based on the fasting condition. This mode of detection does not accurately reflect the level of lipids. In 1979, Zilversmit first proposed that postprandial lipemia was associated with an increased risk of atherosclerosis [1].This finding attracted growing attention in postprandial lipid metabolism and confirmed by others [2, 3]. At present, postprandial lipid disturbance have been seen in persons with obesity [4–6], impaired glucose tolerance [7], first degree relatives of type 2 diabetes families [8], and type 2 diabetes [9–13].

Type 2 diabetes is a global epidemic that poses an immense medical challenge to health-care systems. Abdominal obesity that accompanies type 2 diabetes is frequently associated with atherogenic dyslipidemia [14]. In addition, lipoprotein lipase (LPL) also plays a pivotal role in lipid homeostasis [15]. The PvuII polymorphisms are found in intron 6 of the LPL gene. This genetic polymorphisms might influence the risk of the appearance of coronary arterial disease [16]. However, the effects of PvuII polymorphisms of LPL on postprandial lipid profiles in response to a liquid high-fat meal have not been reported so far in type 2 diabetic patients with abdominal obesity.

Therefore, the aims of this study were to evaluate the postprandial lipid responses after the ingestion of a liquid high-fat meal in type 2 diabetic patients with abdominal obesity, and determine if the PvuII polymorphisms of LPL influence their postprandial lipid metabolism.

## Methods

### Subjects

**Fig. 1:**
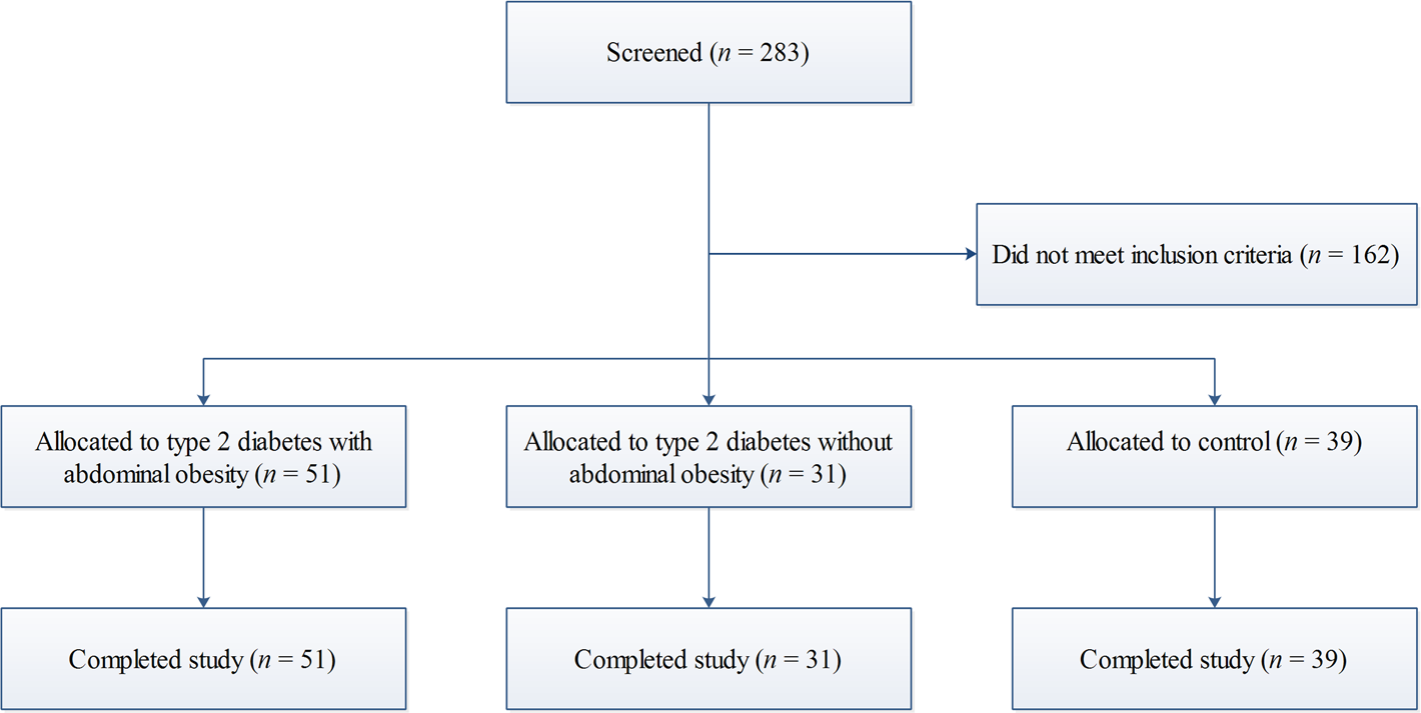
Study flow chart of participant selection

The study was conducted in 51 type 2 diabetic patients with abdominal obesity and 31 type 2 diabetic patients without abdominal obesity, recruited from the Nanjing Jiangpu People Hospital. A group of 39 non-diabetic, non-abdominal obesity controls of similar age was also included (Fig. 1). The sample size was estimated after fixing α value at 0.05 and margin of error at 0.5 (SD = 1.3) for triglyceride (TG) by PASS 11. Diagnosis of type 2 diabetes was based on World Health Organization criteria [17]. Abdominal obesity was defined by Working Group on Obesity of China criteria (waistline ≥85 cm for male and ≥80 cm for female) [18]. All subjects had normal fasting TG level (< 1.7 mmol/L), normal thyroid, hepatic and renal functions. They were not taking any drug known to influence lipid metabolism. The study protocol was approved by the ethic committee of Zhongda hospital affiliated to Southeast University, and written informed consent was obtained from each participant before being tested.

### Study protocol

After a 10–12 h overnight fast, fasting venous blood samples were gathered by an indwelling needle fitted in the subjects’ cubital veins. Following this, a liquid high-fat meal was given to be ingested in 5 min. The 1000 ml test drink was formulated by adding casein 72 g, sucrose 156 g, lactose 12 g, butter 132 g, cholesterol 1.32 g and 7 g monostearin. Its manufacturing process involves heating, mixing, shearing, homogenizing, packaging and autoclaving. The corresponding caloric intake was 20 kcal/kg body weight, 56.9% derived from fat (31.9% saturated fatty acids, 22.0% monounsaturated fatty acids, and 3.0% polyunsaturated fatty acids), 30.5% from carbohydrate, and 12.6% from protein. Preparation of the liquid high-fat meal took place in Taizhou Weigang dairy food corporation under the supervision of Nanjing Municipal Center for Disease Control and Prevention. Subjects were prohibited from strenuous exercise and permitted to consume only water throughout the postprandial period. All subjects were able to eat the entire test meal. At baseline and 0.5, 1, 2, 4, 6 and 8 h after the meal, serum glucose, insulin, TG, total cholesterol (TC) and high density lipoprotein cholesterol (HDL-C) were determined. The PvuII polymorphisms of LPL were tested only in fasting.

### Measurements

Waistline, weight and height were measured according to standardized protocols. Serum glucose, TG, TC and HDL-C were performed by automatic biochemical analyzer (Beckman, DxC800, USA). Serum insulin was determined by chemiluminescence (Roche, FG_cobase 8000, Switzerland). The insulin resistance was estimated using homeostasis model assessment-insulin resistance (HOMA-IR) formula [19]. The postprandial areas under the curve (AUC) was calculated using GraphPad Prism 5. The PvuII polymorphisms of LPL were performed by DNA extraction, polymerase chain reaction, and PvuII restriction enzyme digestion of the amplified products.

### Statistical analysis

Data are expressed as means ± SD, unless otherwise stated. For comparison among groups, analysis of variance (ANOVA) with post hoc test were used. For comparison overtime, repeated measures ANOVA were used. Variables not normally distributed were analyzed after logarithmic transformation or by nonparametric tests. Chi-square test was used for categorical variables. Statistical analysis were performed in PASW statistics 18.0. A value of *P* < 0.05 was considered as statistically significant.

## Results

### Baseline characteristics

The baseline characteristics of all participants are summarized in Table 1. The three groups did not differ by age, sex ratio and PvuII phenotypes. Duration of diabetes, diabetes treatment, glucose, TG, TC and HDL-C were similar in type 2 diabetic patients with and without abdominal obesity. Waistline, body mass index, insulin and HOMA-IR were significantly higher (all *P* < 0.05) in type 2 diabetic subjects with abdominal obesity than those without abdominal obesity and controls.

**Table 1:** Baseline characteristics of all participants

Variables	T2D + AO (*n* = 51)	T2D-AO (*n* = 31)	Control (*n* = 39)
Age, years	56.6 ± 7.5	53.4 ± 9.2	52.8 ± 9.3
Male / female, n	29 / 22	16 / 15	21 / 18
Duration of diabetes, years (median, IQR)	4.0 (2.0–8.0)	3.0 (2.0–5.5)	−
Diabetes treatment, n			
Diet only	3	2	−
Oral agents	38	24	−
Insulin	4	2	−
Insulin + oral agents	6	3	−
Waistline, cm	91.7 ± 5.9 ^a, b^	76.6 ± 4.8	76.6 ± 4.9
BMI, kg/m^2^	25.2 ± 2.7 ^a, b^	21.1 ± 2.0	21.7 ± 2.1
Glucose, mmol/L	7.89 ± 2.06 ^a^	7.74 ± 2.71 ^c^	5.07 ± 0.41
Insulin, uIU/mL (median, IQR)	11.35 (9.42–16.07) ^a, b^	8.57 (6.59–11.77) ^c^	6.93 (5.06–9.24)
HOMA-IR (median, IQR)	4.27 (3.06–5.97) ^a, b^	2.80 (1.93–4.09) ^c^	1.51 (1.15–2.08)
TG, mmol/L	1.11 ± 0.34 ^a^	0.79 ± 0.35	0.77 ± 0.31
TC, mmol/L	4.80 ± 0.90 ^a^	4.66 ± 0.81	4.32 ± 0.85
HDL-C, mmol/L	1.24 ± 0.28 ^a^	1.45 ± 0.30	1.49 ± 0.36
PvuII phenotypes, n			
P+/+	31	16	14
P+/−	16	11	19
P−/−	4	4	6

All data are means ± SD unless otherwise stated. T2D + AO, type 2 diabetes with abdominal obesity; T2D-AO, type 2 diabetes without abdominal obesity; IQR, interquartile range; BMI, body mass index; HOMA-IR, homeostasis model assessment-insulin resistance; TG, triglyceride; TC, total cholesterol; HDL-C, high density lipoprotein cholesterol. ^a^ *P* < 0.05, T2D + AO vs control, ^b^ *P* < 0.05, T2D + AO vs T2D-AO, ^c^ *P* < 0.05, T2D-AO vs control

### Postprandial glycemic status

**Fig. 2:**
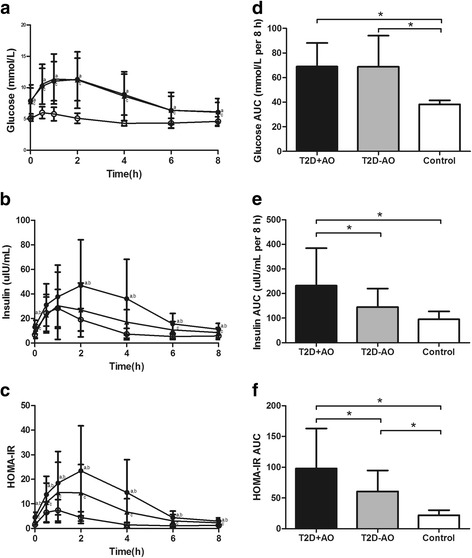
Postprandial glycemic status in different study groups. Left panel: Serum (**a**) glucose, (**b**) insulin and (**c**) HOMA-IR levels over the postprandial period in different study groups. Plotted values are means ± SD. ●, T2D + AO (*n* = 51); ▲, T2D-AO (*n* = 31); ○, Control (*n* = 39). ^a^ *P* < 0.05, T2D + AO vs control, ^b^ *P* < 0.05, T2D + AO vs T2D-AO, ^c^ *P* < 0.05, T2D-AO vs control. For the serum glucose, insulin and HOMA-IR responses, there was a significant time effect (*P* < 0.05), group effect (*P* < 0.05) and group × time interaction (*P* < 0.05) by repeated-measures ANOVA. Right panel: Postprandial (**d**) glucose, (**e**) insulin and (**f**) HOMA-IR AUCs in different study groups. Bars represent means ± SD. ^*^ *P* < 0.05. T2D + AO, type 2 diabetes with abdominal obesity; T2D-AO, type 2 diabetes without abdominal obesity; HOMA-IR, homeostasis model assessment-insulin resistance; AUC, areas under the curve

For the serum glucose, insulin and HOMA-IR responses, there was a significant time effect (*P* < 0.05), group effect (*P* < 0.05) and group × time interaction (*P* < 0.05). In type 2 diabetic patients with abdominal obesity, glucose levels increased between 0.5 and 2 h, in concomitance with the increase in insulin and HOMA-IR (Fig. 2a–c). Glucose levels showed a peak at 0.5 h in controls and at 1 h in type 2 diabetic patients without abdominal obesity (Fig. 2a), insulin and HOMA-IR levels rose significantly in both groups with peak at 1 h (Fig. 2b–c).

Type 2 diabetic patients with abdominal obesity had significantly higher postprandial glucose AUC (LSMD = 30.763, 95% CI = 23.071–38.455, *F* = 37.346, *P* < 0.05) than controls (Fig. 2d). Type 2 diabetic patients with abdominal obesity had higher postprandial AUCs for insulin (LSMD = 86.987, 95% CI = 37.421–136.553, *F* = 16.739, *P* < 0.05) and HOMA-IR (LSMD = 37.456, 95% CI = 16.312–58.600, *F* = 27.012, *P* < 0.05) than those without abdominal obesity (Fig. 2e–f).

### Postprandial lipid status

**Fig. 3:**
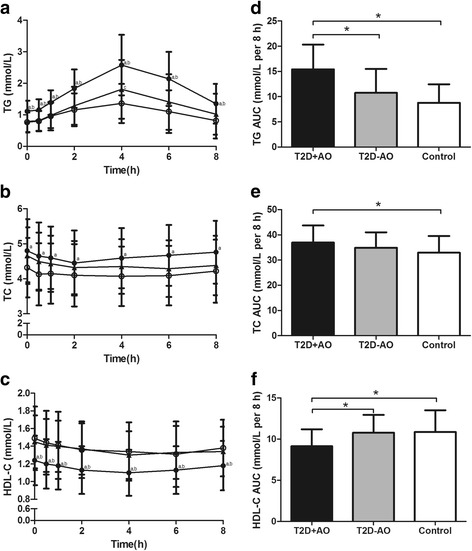
Postprandial lipid status in different study groups. Left panel: Serum (**a**) TG, (**b**) TC and (**c**) HDL-C levels over the postprandial period in different study groups. Plotted values are means ± SD. ●, T2D + AO (*n* = 51); ▲, T2D-AO (*n* = 31); ○, Control (*n* = 39). ^a^ *P* < 0.05, T2D + AO vs control, ^b^ *P* < 0.05, T2D + AO vs T2D-AO, ^c^ *P* < 0.05, T2D-AO vs control. For the serum TG and TC responses, there was a significant time effect (*P* < 0.05), group effect (*P* < 0.05) and group × time interaction (*P* < 0.05) by repeated-measures ANOVA. For the serum HDL-C responses, there was a significant time effect (*P* < 0.05) and group effect (*P* < 0.05) by repeated-measures ANOVA. Right panel: Postprandial (**d**) TG, (**e**) TC and (**f**) HDL-C AUCs in different study groups. Bars represent means ± SD. ^*^ *P* < 0.05. T2D + AO, type 2 diabetes with abdominal obesity; T2D-AO, type 2 diabetes without abdominal obesity; TG, triglyceride; TC, total cholesterol; HDL-C, high density lipoprotein cholesterol; AUC, areas under the curve

For the serum TG and TC responses, there was a significant time effect (*P* < 0.05), group effect (*P* < 0.05) and group × time interaction (*P* < 0.05). For the serum HDL-C responses, there was a significant time effect (*P* < 0.05) and group effect (*P* < 0.05). In type 2 diabetic patients with abdominal obesity, TG levels reached peak concentration at 4 h, and had not returned to fasting concentration at 8 h (Fig. 3a). A significant reduction was observed in TC and HDL-C levels when compared with their fasting concentration (Fig. 3b–c). In both controls and type 2 diabetic patients without abdominal obesity, TG levels peaked at 4 h and had returned to fasting concentration at 8 h (Fig. 3a), TC and HDL-C levels followed a decreasing trend over time (Fig. 3b–c).

Postprandial TG AUC (Fig. 3d) was higher (LSMD = 4.684, 95% CI = 2.662–6.705, *F* = 26.158, *P* < 0.05), whereas HDL-C AUC (Fig. 3f) was lower (LSMD = −1.652, 95% CI = −2.685 – -0.620, *F* = 8.190, *P* < 0.05) in type 2 diabetic patients with abdominal obesity than those without abdominal obesity. Type 2 diabetic patients with abdominal obesity had significantly higher postprandial TC AUC (LSMD = 3.995, 95% CI = 1.043–6.947, *F* = 3.681, *P* < 0.05) than controls (Fig. 3e).

### The influence of PvuII polymorphisms on postprandial glycemic and lipid status

We compared glycemic and lipid responses in type 2 diabetic subjects with abdominal obesity according to PvuII phenotypes. The three phenotypic groups did not differ in demographic characteristics, fasting glycemic and lipid parameters (Table 2).

**Table 2:** Baseline characteristics of type 2 diabetic patients with abdominal obesity according to PvuII phenotype

Variables	P+/+ (*n* = 31)	P+/− (*n* = 16)	P−/− (*n* = 4)
Age, years	56.2 ± 7.8	57.1 ± 7.5	56.3 ± 7.6
Male / female, n	18 / 13	9 / 7	2 / 2
Duration of diabetes, years (median, IQR)	5.0 (2.0–9.0)	4.0 (1.0–4.5)	3.5 (2.5–4.5)
Diabetes treatment, n			
Diet only	2	1	0
Oral agents	22	12	4
Insulin	3	1	0
Insulin + oral agents	4	2	0
Waistline, cm	90.9 ± 5.6	92.8 ± 6.8	90.3 ± 3.8
BMI, kg/m^2^	25.3 ± 2.7	25.4 ± 2.5	25.5 ± 4.6
Glucose, mmol/L	7.98 ± 1.89	7.52 ± 2.39	8.17 ± 2.30
Insulin, uIU/mL (median, IQR)	11.35 (9.59–15.56)	11.95 (9.45–18.10)	10.58 (8.05–11.55)
HOMA-IR (median, IQR)	4.31 (3.19–5.68)	4.21 (2.85–6.16)	3.61 (2.56–4.68)
TG, mmol/L	1.22 ± 0.35	1.16 ± 0.29	0.78 ± 0.35
TC, mmol/L	4.83 ± 0.98	4.76 ± 0.82	4.47 ± 0.80
HDL-C, mmol/L	1.22 ± 0.29	1.27 ± 0.28	1.11 ± 0.13

All data are means ± SD unless otherwise stated. *IQR* interquartile range, *BMI* body mass index, *HOMA-IR* homeostasis model assessment-insulin resistance, *TG* triglyceride, *TC*, total cholesterol, *HDL-C* high density lipoprotein cholesterol

**Fig. 4:**
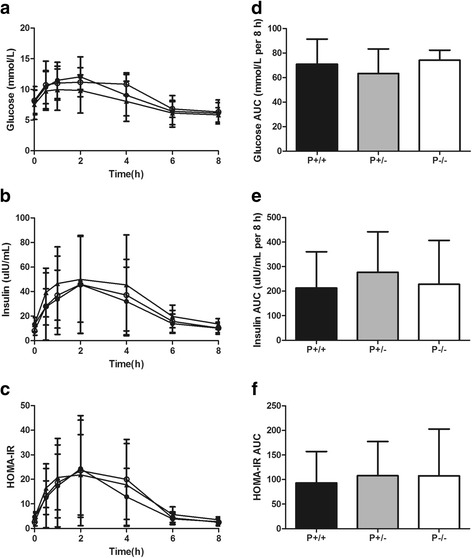
The influence of PvuII polymorphisms on postprandial glycemic status. Left panel: Serum (**a**) glucose, (**b**) insulin and (**c**) HOMA-IR levels over the postprandial period in type 2 diabetic patients with abdominal obesity according to PvuII phenotypes. Plotted values are means ± SD. ●, P+/+ (*n* = 31); ▲, P+/− (*n* = 16); ○, P−/− (*n* = 4). For the serum glucose, insulin and HOMA-IR responses, there was a significant time effect (*P* < 0.05) by repeated-measures ANOVA. Right panel: Postprandial (**d**) glucose, (**e**) insulin and (**f**) HOMA-IR AUCs in type 2 diabetic patients with abdominal obesity according to PvuII phenotypes. Bars represent means ± SD. HOMA-IR, homeostasis model assessment-insulin resistance; AUC, areas under the curve

**Fig. 5:**
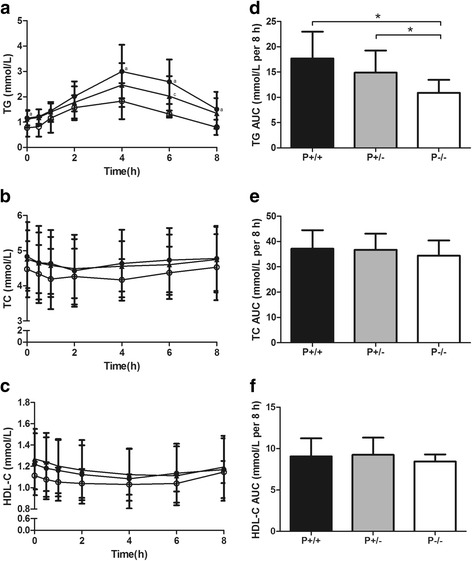
The influence of PvuII polymorphisms on postprandial lipid status. Left panel: Serum (**a**) TG, (**b**) TC and (**c**) HDL-C levels over the postprandial period in type 2 diabetic patients with abdominal obesity according to PvuII phenotypes. Plotted values are means ± SD. ●, P+/+ (*n* = 31); ▲, P+/− (*n* = 16); ○, P−/− (*n* = 4). ^a^ *P* < 0.05, P+/+ vs P−/−, ^b^ *P* < 0.05, P+/+ vs P+/−, ^c^ *P* < 0.05, P+/− vs P−/−. For the serum TG, TC and HDL-C responses, there was a significant time effect (*P* < 0.05) by repeated-measures ANOVA. Right panel: Postprandial (**d**) TG, (**e**) TC and (**f**) HDL-C AUCs in type 2 diabetic patients with abdominal obesity according to PvuII phenotypes. Bars represent means ± SD. ^*^ *P* < 0.05. TG, triglyceride; TC, total cholesterol; HDL-C, high density lipoprotein cholesterol; AUC, areas under the curve

After the liquid high-fat meal, glucose, insulin and HOMA-IR levels were not different according to PvuII phenotypes (Fig. 4a–c). TG levels were lower in P−/− than in P−/+ and P+/+ patients, with the difference reaching significance at 4 h (Fig. 5a). TC and HDL-C levels were lower in P−/− than in P−/+ and P+/+ patients, but this difference did not reach significance (Fig. 5b–c). The postprandial TG AUC was lower in P−/− than in P+/− (LSMD = −4.393, 95% CI = −9.278 – -0.491, *F* = 4.476, *P* < 0.05) and P+/+ (LSMD = −7.180, 95% CI = −12.319 – -2.014, *F* = 4.476, *P* < 0.05) patients (Fig. 5d), whereas postprandial AUCs for glucose, insulin, HOMA-IR (Fig. 4d–f), TC and HDL-C (Fig. 5e–f) were not different in the three phenotypic groups.

## Discussion

In this study, we assess the postprandial lipid responses after a high-fat challenge in type 2 diabetic patients with abdominal obesity, and determine whether the PvuII polymorphisms of LPL influence their postprandial lipid metabolism. As expected, type 2 diabetic patients with abdominal obesity had higher postprandial AUCs for insulin and HOMA-IR than those without abdominal obesity and controls, suggesting greater degree of insulin resistance. Type 2 diabetic patients with abdominal obesity, even with fasting normotriglyceridaemia, showed higher postprandial TG AUC and lower postprandial HDL-C AUC than those without abdominal obesity and controls. Moreover, in type 2 diabetic patients with abdominal obesity, the patients with at least one P+ allele had a greater TG AUC than P−/− patients.

After the high-fat load, we observed a significant TG increase at 4 h in both type 2 diabetic patients and controls. This finding concurs with most previous studies [5, 7, 9, 11, 20, 21]. However, only type 2 diabetic patients with abdominal obesity showed greater TG level after 8 h, thus indicating a decreased TG-clearing capacity. In addition, postprandial TG AUC was not different from controls in type 2 diabetic patients without abdominal obesity. Similar results have been previously observed in non-obese type 2 diabetic patients [22, 23]. The postprandial TG increase in all groups was mirrored by a concomitant decrease in HDL-C. Previous studies conducted in normolipemic [24] and hypercholesterolemic [25] postmenopausal women also found a significant decrease in HDL-C after ingestion of an oral fat load. Interestingly, although postprandial TC AUC was higher in type 2 diabetic subjects with abdominal obesity than controls, a decreased TC levels have also been observed in all groups when compared with their fasting concentration. Reasons for this phenomenon may be that high level of dietary cholesterol suppress the production of cholesterol in erythrocytes [26]. This finding confirms previous studies carried out in diabetic obese [20] and normotriglyceridemic subjects [6]. An important thing to note is that postprandial AUCs for TG, TC and HDL-C were not statistically significant between type 2 diabetic patients without abdominal obesity and controls. Thus, type 2 diabetes by itself does not seem to be sufficient to cause deterioration of postprandial lipid profiles.

LPL is a key enzyme of lipid metabolism, its primary function is to provide free fatty acids and glycerol for energy utilization and storage [27]. Several mutations in the LPL gene will have influence on catabolism of lipoproteins. Recent data suggest that the PvuII polymorphisms of LPL play critical roles in the development of metabolic and cardiovascular disease [28, 29]. However, the influences of PvuII polymorphisms on postprandial lipemia in type 2 diabetic patients with abdominal obesity have not been reported to date. There are, to our knowledge, only 1 previous study assessed association of PvuII polymorphisms of LPL with lipid metabolism in type 2 diabetic patients [30]. This study displayed a higher TG level in P+/+ subjects, indicating a modulating role of P allele on lipid profile. It is likely that the difference would have been more evident after a high-fat meal. The HindIII variant is another characterized polymorphisms of LPL. Pirro et al. [24] assessed the role of HindIII polymorphisms of LPL in hyperlipemic postmenopausal women and found that TG AUC was significantly higher in H+ allele carriers than in subjects with H−/− genotype. In our study, the distribution of PvuII genotypes did not differ in the three groups. However, the patients with at least one P+ allele had a greater postprandial TG AUC than P- patients. It is especially noteworthy that P−/− group was limited to a few samples, which mostly caused by the low frequency in Chinese populations. Additional data are needed to clarify the contribution of PvuII polymorphisms of LPL to postprandial lipemia.

Our study had several limitations. First, the main results of this study reflect an acute postprandial response which may not predict what happens chronically. Second, there are too few subjects with P−/− to be able to draw firm conclusions. Third, no data are available regarding hunger, fullness, and desire to eat collected on the subjects. Finally, results from a selective group cannot be assumed to apply to the total diabetic population.

To study postprandial lipemia, a variety of fat loading tests such as different food type, total food intake, fat content and time points for blood sample collections have been used, which make it difficult to compare results across studies. Here we use a liquid test meal as proposed by Schrezenmeir et al. [31]. It is proved to be fast and simple, and can eliminate the metabolic difference caused by the time of chewing food and mastication masticatory performance. In addition, the amount of food intake is adjusted to Dietary Guidelines for Chinese Residents, based on body weight rather than body surface area. The corresponding contribute rate of fat in total energy was 56.9%, with similar proportion in several other studies [5, 7, 20, 21, 32]. This approach not only brings efficiency with respect to calculation but also satisfies the various energy requirements of subjects. Moreover, blood collection in fasting and postprandial state at 0.5, 1, 2, 4, 6 and 8 h can accurately reveal the postprandial state of lipid.

## Conclusions

In summary, abdominal obesity exacerbates the postprandial lipid responses in type 2 diabetic patients, which partly explains the excess atherogenic risk in these patients. In addition, the presence of P+ allele could contribute to a greater postprandial TG increase in type 2 diabetic patients with abdominal obesity. Further studies are required to elucidate the mechanisms responsible for the altered postprandial lipid profiles in type 2 diabetic patients with abdominal obesity.

## Abbreviations

AUC: Areas under the curve
CI: Confidence interval
HDL-C: High density lipoprotein cholesterol
HOMA-IR: Homeostasis model assessment-insulin resistance
IQR: Interquartile range
LPL: Lipoprotein lipase
LSMD: Least square mean difference
TC: Total cholesterol
TG: Triglyceride
